# A case-control retrospective study for the effect of Shoutai Wan on IVF-ET pregnancy outcome

**DOI:** 10.1097/MD.0000000000036846

**Published:** 2024-01-05

**Authors:** Qing Liu, Chenyun Miao, Fangxuan Lin, Han Zhang, Qin Zhang

**Affiliations:** a Zhengjiang Chinese Medical University, Hangzhou, China; b Department of Traditional Chinese Medicine gynecology, The Hangzhou Hospital of Zhejiang Chinese Medical University, Hangzhou, China.

**Keywords:** Shoutai Wan IVF-ET pregenancy miscarriage

## Abstract

It has a long history of preventing and treating disease using traditional Chinese medicine (TCM). In recent years, it has been widely used in adjuvant therapies of in vitro fertilization - embryo transfer (IVF-ET) in China. To observe the effect and safety of Shoutai Wan on pregnancy outcomes after IVF-ET. A total of 352 patients who underwent IVF-ET from July 1, 2020 to June 30, 2021. The participants who only received routine luteal support during clinical pregnancy of FET were defined as the control group, and others who received TCM decoction Shoutai Wan for prevention of miscarriage and routine luteal support were defined as the Chinese medicine protection Shoutai Wan group (St group). This project has been approved by the Ethics Committee of Run Run Shaw Hospital Affiliated to Zhejiang University School of Medicine (Approval NO: 2023-0305). The results of this retrospective cohort study revealed that Shoutaiwan combined with luteal support treatment can significantly decreased the miscarriage rate in pregnancy undergoing IVF-FET compared to the group accepted only luteal support treatment (*P* = .001). Meanwhile, St during pregnancy did not affect fetal birth weight (*P* = .354), and there was no adverse event in the St group reported, which confirmed the safety of TCM for fetus protection during pregnancy. This study not only provides evidences for the clinical administration of Shoutai Wan in IVF-ET, but also provides a novel direction for basic research into the subsequent innovative application of TCM.

## 1. Introduction

According to the World Health Organization, infertility afflicts about 15% of couples at reproductive age worldwide, which equals about 48 to 72 million couples. Since 1978, in vitro fertilization and embryo transfer (IVF-ET) has been an important treatment for infertility. It is however unfortunate that IVF-ET is not as successful as expected.^[2]^ Study has shown that IVF-ET patients experience recurrent implantation failures in about 15% of cases.^[3]^ In clinical practice, symptoms of threatened abortion (TA) such as vaginal bleeding and abdominal pain are very common during pregnancy of IVF-ET women. TA is defined as vaginal bleeding through a closed cervical so during the first 20 weeks of pregnancy with or without abdominal pain, which has the potential to develop into a miscarriage. Therefore, effective preventive measures is urgently needed.

It has a long history of preventing and treating disease using traditional Chinese medicine (TCM). In recent years, it has been widely used in adjuvant therapies of IVF-ET in China. Shoutai Wan (St) is a classic prescription by Xichun Zhang of the Qing Dynasty. Its pharmaceutical composition consists of Cuscuta sinensis, Sambucus nigra, Dipsaci Radix, and Agaricus blazei.^[4]^ Based on evidence indicating that St is effective for prevention of miscarriage in the first trimester of pregnancy women with unexplained recurrent spontaneous abortion.^[5]^ However, there is currently no clinical evidence for the specifics of St in the treatment of TA in IVF-ET patients.

Hence, we conducted a retrospective cohort study with large sample based on the clinical data from the center for reproductive medicine of the Sir Run Run Shaw Hospital in the east of China. This study aimed to investigate the effect of St on the pregnant outcomes in patients undergoing IVF-ET.

## 2. Materials and methods

### 2.1. Research subjects and grouping

Clinical data of this study were collected from electronic medical records in the Reproductive Center, Sir Run Run Shaw Hospital affiliated with the Zhejiang University School of Medicine.

#### 2.1.1. Inclusion criteria.

(i) Age of oocyte retrieval ≤ 40 years old.
(ii) No embryo transfer was performed before. This was the first time for thawed embryo transfer, and 2 high-quality embryos were transplanted in all cases.
(iii) At least 2 embryos (embryos at cleavage stage or blastocyst stage) remained in cryopreservation, and there was ≥ 1 high-quality embryo.

#### 2.1.2. Exclusion criteria.

(i) Patients with endometrial thickness ≤ 8 mm detected by B-mode ultrasound of the implant window with presence of endometrial polyps, intrauterine adhesions, intrauterine effusion, and hydrosalpinx.
(ii) Patients with endocrine and metabolic disorders, thrombophilia, antiphospholipid syndrome, Prethrombotic state, autoimmune diseases, etc.
(iii)  < 2 remaining embryos.
(iv) Patients who were on TCMs except modified St.

### 2.2. Treatment

frozen embryo transfer (FET) patients with hormone replacement cycle had oral estradiol valerate tablets (DELPHARM Lille S.A.S, France) at 3 mg twice daily starting from the 3^rd^ day of menstruation; After 1 week of medication, estradiol valerate tablets were increased to 4 mg each time, twice a day for about 1 week; Endometrial transformation was performed when endometrial thickness was ≥ 8 mm, and blood estrogen (E2) and progesterone (Pg) levels were measured at the same time. FET was performed on the 3^rd^ day after endometrial transformation. Luteal support protocol for hormone replacement cycle was progesterone soft capsules (Zhejiang Aisheng Pharmaceutical Co., Ltd, China) administered vaginally, 200 mg each time, twice a day; Dydrogesterone tablets (Abbott B.V., German) were taken orally, 20 mg, twice a day. During the clinical pregnancy of FET, the conventional protocol group only received conventional luteal support, and the St group received TCM decoction containing Shoutai Wan for fetus protection and conventional luteal support at the same time after FET clinical pregnancy. Shoutai Wan consists of Semen Cuscutae, Herba Taxilli, Dipsaci Radix, and Asini Corii Colla (Table 1). Above crude herbs were purchased from the Hangzhou Hospital of Zhejiang Chinese Medical University. The decoctions were prepared through the addition of 1 dose (50 g medicinal herbs) to 400 mL of water, followed by boiling for a duration of 1 hour, 1 dose/day, taken in 2 servings.

**Table 1:** Information of Shoutai Wan.

	Local Name	Latin Name	Family Name	English Name	Part used	Amount (g)
1	Tu-Si-Zi	*Cuscuta chinensis* Lam.	Convolvulaceae	Semen Cuscutae	Seed	20
2	Sang-Ji-Sheng	*Taxillus sutchuenensis* (Lecomte) Danser	Mulberry parasitic plants	Herba Taxilli	Stem	10
3	Chuan-Xu-Duan	*Dipsacus asper* Wall.ex Henry	Dipsacaceae	Dipsaci Radix	Root	10
4	E-Jiao	*Equus asinus* L.	Equidae	Asini Corii Colla	Solid glue	10

### 2.3. Outcome measures

The number of miscarriages after FET in the 2 groups and the time of taking TCM in the St group were counted, and the miscarriage rate of FET after hormone replacement in the 2 groups was calculated, as well as the adverse effects and prognosis. Miscarriage rate = total number of cycles miscarried/total number of clinical pregnancy cycles × 100%. Common adverse effects are abnormal liver function, abnormal kidney function, fever, rash, ectopic pregnancy, and embryo growth arrest, etc. A gestational sac and fetal heart seen ultrasonographically 50 days after transplantation were defined as clinical pregnancy.

### 2.4. Statistical analyzes

SPSS 19.0 software was used for data analysis. Measurement data were expressed as mean ± standard deviation (x ± s), and comparison was performed by *t test* or variance analysis; The statistical data were expressed as rate (%), comparisons were made using *χ^2^* test. α = 0. 05 was used as the test level.

## 3. Results

In this study, 352 patients underwent FET from July 1, 2020 to June 30, 2021 at the Reproductive Center of Sir Run Run Shaw Hospital affiliated with the Zhejiang University School of Medicine.

### 3.1. Baseline characteristics of enrolled cases

There were no significant differences in age at oocyte retrieval as shown by the following table (Table 2), years of infertility, antimullerian hormone, body mass index, endometrial thickness and morphology on the day of transplantation, or the level of estrogen and progesterone on the day of transplantation between the 2 groups (*P* > .05).

**Table 2:** Baseline characteristics of enrolled cases.

	St M (P_25_,P_75_)	Control M (P_25_,P_75_)	Wilcoxon z	*P* value
Age (yr)	31 (29–33)	31 (29–34)	0.187	.852
BMI (kg/m^2^)	21.3 (19.4–23.4)	21.5 (19.9–24.2)	1.184	.237
AMH (ug/mL)	2.5 (1.7–6.8)	2.8 (1.5–4.3)	1.078	.281
Endometrial thickness on transplantation d (mm)	9.5 (8.5–10.6)	9.8 (8.7–10.6)	0.990	.332

AMH = antimullerian hormone, BMI = body mass index, St = Shoutai Wan.

### 3.2. Shoutai Wan reduced miscarriage rate in patients undergoing first IVF-FET

Chi square test was used to analyze the effect of the conventional protocol group or the TCM fetus protection group on miscarriage, as shown by the following table (Table 3), when the significance level *P* < .05, it can be considered that the use of TCM fetus protection has a significant difference in miscarriage.

**Table 3:** The miscarriage rate of control and St groups.

	Normal pregnancy (n[%])	Miscarriage (n[%])	Chi-square value	*P* value
Control (n)	202 (85.23%)	35 (14.77%)	11.887	.001
St (n)	112 (97.39%)	3 (2.61%)		

St = Shoutai Wan.

### 3.3. Shoutai Wan had no effect on the birth weight of the fetus

Since the significance level of 0.05 that we can see from the analysis results in the following table (Table 4), it can be considered that there is no significant difference in fetal weight between the groups with or without the use of TCM.

**Table 4:** The birth weight of the fetus of control and St groups.

Groups	N	Birth weight of the fetus (x ± s, g)	*t* value	*P* value
Control	202	3053.63 ± 613.80	0.928	.354
St	112	2984.24 ± 669.86		

St = Shoutai Wan.

### 3.4. Adverse effects and prognosis

None of the included patients in both groups had obvious adverse effects such as abnormal liver function, abnormal kidney function, fever, rash, ectopic pregnancy, embryo growth arrest, etc.

## 4. Discussion

Based on the TCM theory, TA is mainly caused by kidney deficiency. Shoutai Wan, recorded in *Yi Xue Zhong Zhong Can Xi Lu* written by the famous doctor Xichun Zhang has the function of supplementing kidney deficiency and stabilizing *Chong Mai* result in the therapy effect on TA. In clinical use, Shoutai Wan is the basis of other prescriptions for TA, and herbs can be added or subtracted according to the syndrome differentiation based on the variable evidence appearing during treatment.^[6]^

As increasingly basic researches emerge, many evidences revealed that the protection of St was related to various biological behavior in maternal-fetal interface. St could enhance the proliferative activity of trophoblastic cells, invasion and migration capacity.^[7]^ The study found St restored balance of Th1/ Th2 cytokines that play an important role in endometrial receptivity and promote embryo implantation.^[8]^ Additionally, metabolic environment is the other factor bridging St and TA. Glycolysis is the main metabolic mode under hypoxic condition, and found to be suppressed in the maternal-fetal interface of kidney deficiency threatened abortion model mice. St improved the level of glycolysis to reduce pregnancy loss rate.^[9]^ Liuting Zeng, et al^[10]^ identified HSP27, α-enolase and Transferrin as the therapeutic targets of St for TA via proteomics strategy and in vivo experiment. The effect of St associated with synaptic transmission, oxidation reduction process, gamma-aminobutyric acid signaling pathway, release of sequestered calcium ion into cytosol, and so on. Above all, we believe that multicompounds, multitargets, and multiple pathways all contributed to the effectiveness of St.

The present study has certain limitations that we must acknowledge. Initially, it was inevitable that bias would be present in the selection of patients for this non-randomized and retrospective study. Further prospective cohort studies or randomized controlled studies with a larger sample size are necessary to establish a higher level of evidence-based evidence regarding the efficacy of TCM in IVF. Additionally, the prescriptions that utilized in the study were subject to modifications based on St, there was significant variation in prescription usage among individuals. Furthermore, evidence-guided medication analysis and outcome correlation analysis were not conducted, which warrants further investigation.

## Acknowledgements

This work was supported by a key project funded by science and technology department of Zhejiang province: the secondary development projects of Jianpi Antai decoction (2021C03080) and National famous and old TCM expert inheritance studio construction project (Guozhong Pharmaceutical Renjiaohan (2022) 75).

## Author contributions

**Data curation:** Qing Liu, Fangxuan Lin, Han Zhang.

**Formal analysis:** Chenyun Miao.

**Methodology:** Qin Zhang.

**Project administration:** Qin Zhang.

**Writing – original draft:** Qing Liu.

**Writing – review & editing:** Qin Zhang.
